# Population genomics through time provides insights into the consequences of decline and rapid demographic recovery through head‐starting in a Galapagos giant tortoise

**DOI:** 10.1111/eva.12682

**Published:** 2018-08-13

**Authors:** Evelyn L. Jensen, Danielle L. Edwards, Ryan C. Garrick, Joshua M. Miller, James P. Gibbs, Linda J. Cayot, Washington Tapia, Adalgisa Caccone, Michael A. Russello

**Affiliations:** ^1^ Department of Biology University of British Columbia Okanagan Kelowna British Columbia Canada; ^2^ Life and Environmental Sciences University of California Merced California; ^3^ Department of Biology University of Mississippi Oxford Mississippi; ^4^ Department of Ecology and Evolutionary Biology Yale University New Haven Connecticut; ^5^ College of Environmental Science and Forestry State University of New York Syracuse New York; ^6^ Galapagos Conservancy Fairfax Virginia; ^7^ Department of Applied Research Galapagos National Park Service Puerto Ayora Ecuador; ^8^ Galapagos Conservancy Santa Cruz Ecuador; ^9^ Present address: Department of Biology Queen's University Kingston Ontario Canada

**Keywords:** bottleneck, *Chelonoidis*, effective population size, historical DNA, hybrid capture, museum specimen, population genetics, RAD‐seq

## Abstract

Population genetic theory related to the consequences of rapid population decline is well‐developed, but there are very few empirical studies where sampling was conducted before and after a known bottleneck event. Such knowledge is of particular importance for species restoration, given links between genetic diversity and the probability of long‐term persistence. To directly evaluate the relationship between current genetic diversity and past demographic events, we collected genome‐wide single nucleotide polymorphism data from prebottleneck historical (c.1906) and postbottleneck contemporary (c.2014) samples of Pinzón giant tortoises (*Chelonoidis duncanensis*;* n *=* *25 and 149 individuals, respectively) endemic to a single island in the Galapagos. Pinzón giant tortoises had a historically large population size that was reduced to just 150–200 individuals in the mid 20th century. Since then, Pinzón's tortoise population has recovered through an ex situ head‐start programme in which eggs or pre‐emergent individuals were collected from natural nests on the island, reared ex situ in captivity until they were 4–5 years old and subsequently repatriated. We found that the extent and distribution of genetic variation in the historical and contemporary samples were very similar, with the latter group not exhibiting the characteristic genetic patterns of recent population decline. No population structure was detected either spatially or temporally. We estimated an effective population size (*N*_e_) of 58 (95% CI = 50–69) for the postbottleneck population; no prebottleneck *N*_e_ point estimate was attainable (95% CI = 39–infinity) likely due to the sample size being lower than the true *N*_e_. Overall, the historical sample provided a valuable benchmark for evaluating the head‐start captive breeding programme, revealing high retention of genetic variation and no skew in representation despite the documented bottleneck event. Moreover, this work demonstrates the effectiveness of head‐starting in rescuing the Pinzón giant tortoise from almost certain extinction.

## 1 INTRODUCTION

A broader understanding of the genetic consequences of population decline is of fundamental importance for species restoration, as standing levels of genetic diversity are associated with the probability of long‐term population persistence (Frankham, 1997, 2005; Frankham et al., 2017), ability to survive novel disease threats (Smith, Acevedo‐Whitehouse, & Pedersen, 2009) and adaptation to changing environments (Barrett & Schluter, 2008; Jump, Marchant, & Peñuelas, 2009). Declining populations often experience genetic bottlenecks, where effective population sizes become very small and the number of allelic variants in the gene pool rapidly diminishes. Many empirical studies have examined the genetic consequences of bottlenecks indirectly, either in natural populations postdecline (for early examples, see O'Brien et al., 1987; Packer et al., 1991) or in experimental settings (Leberg, 1992). Most studies have focused on the decline phase of bottlenecks (e.g., England et al., 2003; Spencer, Neigel, & Leberg, 2000), while far fewer have examined the recovery phase. Previously, there were few examples of direct investigations of changes in genetic variation in natural populations before and after a known bottleneck event. When studies employed temporally spaced sampling, they typically relied on a limited number of genetic markers to characterize population‐level patterns of genetic diversity (e.g., a fragment of the mitochondrial DNA control region and/or 5–24 microsatellite loci; Bouzat, Lewin, & Paige, 1998; Eldridge et al., 2004; Miller & Waits, 2003; Nyström, Angerbjörn, & Dalén, 2006; Ugelvig, Nielsen, Boomsma, & Nash, 2011; Wisely, Buskirk, Fleming, McDonald, & Ostrander, 2002). In recent years, however, there are a growing number of temporal studies investigating bottlenecks using full mitochondrial genome sequences (e.g., Dussex, von Seth, Robertson, & Dalén, 2018; Jensen et al., 2018; van der Valk et al., 2018) and genome‐wide markers (e.g., Der Sarkissian et al., 2015; Mikheyev, Tin, Arora, & Seeley, 2015).

**Figure 1:**
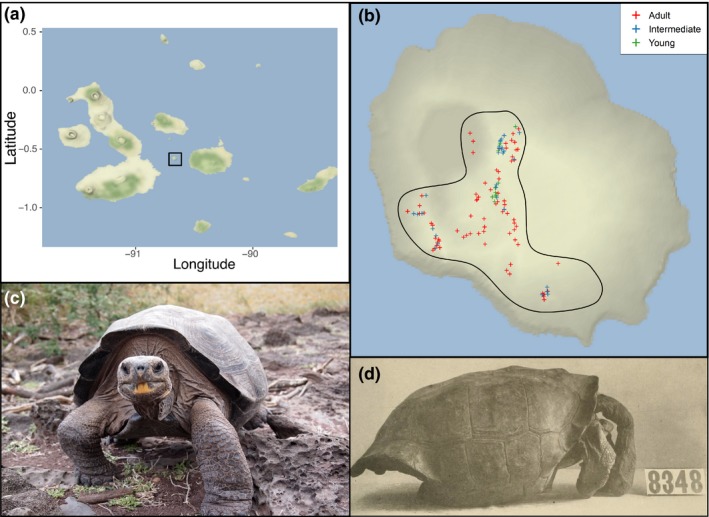
Maps and images of Pinzón giant tortoises. (a) The Galapagos Archipelago, with the black box indicating Pinzón Island. (b) Inset of Pinzón Island, with sampling locations for contemporary individuals indicated. The outline indicates the areas on the island with suitable habitat for tortoises. The coloured symbols represent the curved carapace length of individuals: Adults are >65 cm, intermediates range from <65 cm to >35 cm, young are <35 cm. (c) An adult repatriated Pinzón giant tortoise (image by E.L. Jensen). (d) A Pinzón giant tortoise specimen in the California Academy of Sciences collection. Image originally from Van Denburgh (1914) and reproduced from the public domain as accessed from the open access Biodiversity Heritage Library (http://www.biodiversitylibrary.org/). Map tiles by Stamen Design, under CC BY 3.0. Data by OpenStreetMap, under ODbL

The Pinzón giant tortoise, *Chelonoidis duncanensis* (previously *Chelonoidis ephippium*; Turtle Taxonomy Working Group, 2017), is endemic to Pinzón Island (18 km^2^ area) in the Galapagos (Figure 1) and consists of a single population. Historically, Pinzón giant tortoises numbered in the thousands, but mass harvesting for food by humans in the early to mid‐1800s dramatically reduced the population size (Townsend, 1931). In the 1890s, black rats (*Rattus rattus*) were introduced to the island and depredated all hatchling tortoises, resulting in no young Pinzón giant tortoises surviving from that time onward (Pritchard, 1996). Surveys conducted in the 1960s located 100 mature *C. duncanensis* on the island and estimated the census population size to be 150–200 individuals, all born before the introduction of rats (MacFarland, Villa, & Toro, 1974). At that point, the Pinzón giant tortoise was a species of “living dead” due to the absence of any recruitment, seemingly destined to become extinct when the last of the remaining ageing adults died.

Faced with the potential loss of the Pinzón giant tortoise, the Charles Darwin Research Station initiated a head‐start programme in 1965, collaboratively managed with the Galapagos National Park Directorate. Eggs or pre‐emergent individuals were collected from natural nests on Pinzón Island and reared in captivity until they were 4–5 years old. Juveniles at this age can avoid predation by rats and were repatriated (Cayot, 2008). The programme successfully repatriated more than 800 juvenile tortoises over the past 50 years. However, unless the cause of hatchling mortality in the wild was addressed, the programme would have to operate in perpetuity (Cayot, 2008). In recognition of this, the Galapagos National Park carried out a rat poisoning campaign in December 2012. In 2014, the first wild‐born hatchlings of *C. duncanensis* were observed in over a century, and the rat eradication project was considered a success (Tapia, Malaga, & Gibbs, 2015). Taken together, these efforts have helped rescue the Pinzón giant tortoise from extinction.

The known history of decline and recovery of *C. duncanensis* provides a rare opportunity to perform a direct evaluation of the relationship between current genetic diversity and past demographic events, including the impact of the head‐start programme on prospects for long‐term survival of the species. Importantly, a large number of museum specimens (*n *=* *86 adults, Figure 1) were collected from Pinzón Island during a 1905–1906 California Academy of Sciences expedition to Galapagos (Van Denburgh, 1914). Given the long lifespan (>100 years) and generation time of Galapagos giant tortoises (25 years, Throp, 1975), coupled with the well‐documented history of decline in the Pinzón species, these museum specimens are likely representative of the prebottleneck population. Thus, they provide a valuable reference point for investigating changes in both the extent and distribution of genetic variation over the past century. Notably, despite the historical specimens being collected more than 100 years ago, only a single generation likely elapsed since their collection due to the “recruitment pause” between the late 1890s and the 1960s. As the historical specimens were collected from a naturally reproducing population, the distribution of genetic variation in that sample can be used to evaluate whether human‐assisted survival of individuals through the head‐start programme has caused a skew in genetic variation.

In this study, we assessed the genetic impacts of a known bottleneck, using pre‐ and postbottleneck samples of Pinzón giant tortoises—a species that has now demographically recovered. We paired restriction‐site‐associated DNA sequencing (RAD‐seq) with targeted capture techniques to collect genome‐wide single nucleotide polymorphism (SNP) genotypic data from historical (prebottleneck) and contemporary (postbottleneck) samples. We then used this SNP data set to directly investigate changes in genome‐wide diversity over time. Additionally, given that the majority of the tortoises in the contemporary sample are the product of the head‐start programme, we evaluated the degree to which genetic variation has been impacted by this conservation intervention.

## 2 MATERIALS AND METHODS

### 2.1 Tissue sample collection and DNA extraction

#### 2.1.1 Contemporary (postbottleneck) samples

Blood samples from 150 individuals (Supporting Information Data S1) were collected over 6 days in December 2014. All parts of the island known to have giant tortoises were surveyed. Each encountered individual was measured along the curved length of its carapace, as carapace length is an indication of age. Each tortoise's geographic location and any distinguishing marks were also recorded. Blood (0.1–1.0 ml) was collected from the brachial artery. Additional details regarding sample collection and DNA extraction can be found in the Supporting Information Supplemental Methods in Appendix S1.

#### 2.1.2 Historical (prebottleneck) samples

Complete adult specimens of *C. duncanensis* had been collected from Pinzón Island in December 1905 through August 1906; details of the collections and the expedition are given in Van Denburgh (1914). Field notes suggest that specimens were collected from throughout the island. Femurs attached to carapaces were sampled from 78 of these specimens accessioned at the California Academy of Sciences (Supporting Information Data S1). All individuals were adults, 57 females and 21 males, with carapace lengths ranging from 53 to 87 cm (Van Denburgh, 1914). DNA was extracted from wedge cuts of bone in a dedicated ancient DNA laboratory at The University of British Columbia Okanagan using a modified version of extraction protocol Y from Gamba et al. (2016) described in the Supporting Information Supplemental Methods in Appendix S1.

### 2.2 Molecular and bioinformatic methods

The detailed information regarding data collection and bioinformatic processing can be found in Supporting Information Supplemental Methods in Appendix S1. Briefly, we used RAD‐seq to simultaneously identify and genotype SNPs in the contemporary sample of Pinzón giant tortoises. We generated RAD libraries for 150 individuals sampled in 2014 using a modified version of Etter, Bassham, Hohenlohe, Johnson, and Cresko's (2011) protocol. The STACKS V1.3 suite of scripts (Catchen, Amores, Hohenlohe, Cresko, & Postlethwait, 2011; Catchen, Hohenlohe, Bassham, Amores, & Cresko, 2013) was used for sequence assembly and SNP discovery. The data set of RAD‐seq loci that were identified by STACKS as having variable sites meeting the filtering criteria (Data S2) was sent to MYcroarray (Ann Arbor, MI, USA) to be used to develop baits to capture these targeted loci in the historical samples. The historical DNA extracts were sent to MYcroarray to construct the libraries and perform hybridization captures. All sequencing was performed on an Illumina HiSeq 2500 at the Yale Center for Genome Analysis.

The reference “genome” used for alignment of nuclear capture loci consisted of 140‐bp‐long target sequences, as well as 100 bp of flanking sequence on either end obtained from a draft genome of *Chelonoidis abingdonii* (Quesada et al., in preparation). Sequences were processed using the BAM pipeline in PALEOMIX (version 1.2.6, Schubert et al., 2014), which employs other standard bioinformatic tools alongside native scripts to support the pipeline. To allow the sequences to be compared between historical captures and contemporary RAD‐seq data, the fastq files retained following the *clone_filter* step in the STACKS workflow were run through PALEOMIX, using the same procedure as for the historical individuals, excluding the DNA damage correction, starting at the mapping stage.

Genotype calling was performed on the combined BAM files generated from the historical and contemporary individuals and from the contemporary individuals alone to produce two data sets: SNPs genotyped in both temporal samples, and a larger pool of SNPs genotyped in the contemporary sample only. We tested several different combinations of mapping and genotype calling approaches on the contemporary individuals to evaluate the impact of different workflows on the outcomes. The details and results are provided in the Supporting Information Supplemental Methods in Appendix S1. Here, we present the analyses used with the data set from the best combination. We implemented stringent filtering of the variable sites and ultimately retained only the first SNP per target region to produce a data set of loci for population genetic analyses. Only individuals with >50% of SNPs genotyped were retained.

We assessed error associated with genotyping of SNP loci by calculating the number of genotype mismatches between two pairs of replicate contemporary individuals (tortoise ID #'s A025 and G154, Supporting Information Data S1) that had been processed independently from DNA extraction onwards.

### 2.3 Population genetic analyses

#### 2.3.1 Within‐population diversity

We used the genotypic data to calculate standard measures of within‐population genetic diversity for the contemporary and historical samples separately, including heterozygosity and *G*_IS_ using GENODIVE V 2.0b27 (Meirmans & Van Tienderen, 2004). Individual inbreeding coefficients were calculated using VCFTOOLS (Danecek et al., 2011). Pairwise relatedness (Queller & Goodnight, 1989) was calculated within the contemporary and historical samples separately using the *Related* package (Pew, Muir, Wang, & Frasier, 2015) in the R statistical package, version 3.2.2 (R Development Core Team, 2015). A genotyping error rate of 4.5% was used (empirically determined, see Supporting Information Table 1 in Appendix S1). Heterozygosity, inbreeding coefficients and pairwise relatedness were calculated using the small (*n *=* *2,218 SNPs, including loci in common among temporal samples) and large (*n *=* *7,730 SNPs, including only loci in the contemporary sample) data sets to assess the impact of the number of loci on the results.

Effective population sizes (*N*_e_) of the contemporary and historical sample groups were calculated using the bias‐corrected measure of linkage disequilibrium (Hill, 1981; Waples, 2006; Waples & Do, 2010), as implemented in NeESTIMATOR V2.1 (Do et al., 2014). We explored the effects of the number of individuals on estimates of *N*_e_ by creating 50 random subsets in increments of 10 individuals, from 10 to 100 individuals, pulled from the contemporary sample group and using both the 2,218 and 7,730 SNP data sets. We estimated *N*_e_ for each subset using a minor allele frequency (MAF) cut‐off of 0.05 and viewed the results in R.

#### 2.3.2 Population substructure analyses

The evidence for substructure within the combined sample (contemporary plus historical individuals) was assessed using Bayesian clustering analysis, as implemented in STRUCTURE 2.3.4 (Pritchard, Stephens, & Donnelly, 2000). Run length was set to 500,000 Markov chain Monte Carlo replicates after a burn‐in period of 100,000 using correlated allele frequencies under an admixture model with alpha set to 0.5. We varied the number of clusters (*K*) from one to four, with ten iterations of each. The most likely number of clusters was determined by plotting the log probability of the data (ln Pr(*X|K*)) across the range of *K* values tested and selecting the *K* where the value of ln Pr(*X|K*) plateaued, as suggested in the STRUCTURE manual.

We also used the model‐free discriminant analysis of principal components (DAPC; Jombart, Devillard, & Balloux, 2010) implemented in *adegenet* (Jombart, 2008) in R. The best‐fit value of *K* was selected using the *find.clusters* function and Bayesian information criterion (BIC). The chosen value of *K* was based on the minimum number of clusters after which the BIC decreased by a negligible amount.

To assess whether there were differences in the genetic diversity captured in the head‐start programme over time, we used carapace size as a proxy for age of sampled individuals in order to divide samples from the contemporary sample group into two age classes and repeated the calculation of diversity metrics (see *Within‐population diversity*, above). The age classes were “adult” samples, those with a curved carapace length >65 cm (*n *=* *82), and “young” samples, those with carapace lengths <35 cm and >15 cm (*n *=* *29). Individuals <15 cm were excluded because they are wild‐born hatchlings that were not part of the head‐start programme. Admittedly, these age classes are somewhat arbitrary, but were chosen to represent nonoverlapping segments of the contemporary sample group that was head‐started either in the early years of the programme (“adults”) or very recently (“young”). This grouping allowed comparisons of levels of diversity within each time point and of the patterns of pairwise relatedness within and among the two temporal sample groups.

The *Phi*_ST_ metric of differentiation was calculated between temporal sample groups in GENODIVE with significance assessed using 999 permutations. Exact tests for differences in allele frequencies between the historical and contemporary sample groups, and between the historical sample and two age classes within the contemporary sample group, were performed in GENEPOP v4.5 (Raymond & Rousset, 1995), with significance assessed using an adjusted *p*‐value based on the correction for false discovery rate described by Benjamini and Yekutieli (2001).

To evaluate evidence for spatial genetic structure within the contemporary sample group, we compared the straight line geographic distance between pairs of individuals at the time of sampling, calculated using the GEOGRAPHIC DISTANCE MATRIX GENERATOR (Ersts, 2012), and their pairwise relatedness. Only individuals with a curved carapace length >50 cm (i.e., mature adults, *n *=* *99) were used in this analysis to exclude recently repatriated cohorts that have not yet had time to disperse away from the release sites (Figure 1b). No spatial data were available for the museum specimens, precluding a similar analysis for the historical sample group.

## 3 RESULTS

We obtained RAD‐seq data from 150 contemporary Pinzón giant tortoises (collected c.2014) representatively sampled from across the island to identify polymorphic SNPs and flanking sequences that could be used to design targeted capture baits for use with the historical samples (collected c.1906). The details of the sequencing output and processing resulting from the RAD‐seq and captures can be found in the Supporting Information Supplementary Results in Appendix S1.

**Figure 2:**
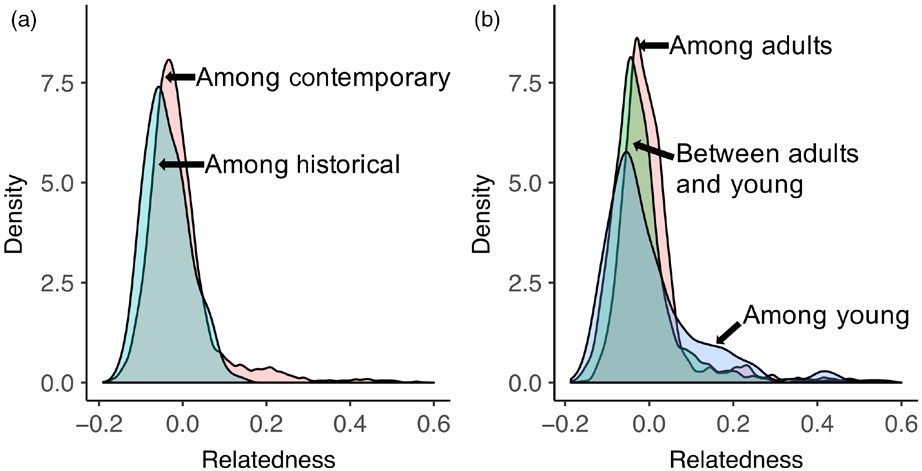
Frequency distributions of pairwise relatedness. Relatedness values are as follows: (a) among individuals within the contemporary and historical sample groups; and (b) among the two size classes “adults” and “young” from the contemporary sample group, and the relatedness values between pairs of “adult” and “young” contemporary individuals. Relatedness estimates were calculated following Queller and Goodnight (1989) based on 2,218 SNP loci. SNP, single nucleotide polymorphism

Our data set for population genetic analysis consisted of 2,218 SNP loci, genotyped in 25 historical and 149 contemporary individuals (Supporting Information Table 2 in Appendix S1, see Supporting Information Supplementary Results in Appendix S1 for genotype quality results). Levels of heterozygosity and inbreeding were very similar in the historical and contemporary sample groups, as was the mean relatedness among individuals (Table 1) and the distributions of pairwise relatedness values within each sample group (Figure 2a).

**Table 1:** Within‐sample group diversity metrics

	Sample	*N*	*H*_o_	*H*_e_	*G*_IS_	*F*	Mean *R*_Q&G_	*N*_e_ (95% CI)
2,218 SNP	Historical	25	0.303	0.285	−0.063	−0.061	−0.037	Infinity
	Contemporary	149	0.305	0.283	−0.077	−0.074	−0.009	58 (50, 69)
	Adult^a^	82	0.297	0.280	−0.059	−0.044	0.005	—
	Young^a^	29	0.320	0.283	−0.129	−0.124	0.004	—
7,785 SNP	Contemporary	149	0.320	0.296	−0.082	−0.083	−0.008	59 (51, 69)

Notes

CI, jackknife confidence interval; *F*, inbreeding coefficient; *G*_IS_, inbreeding coefficient; *H*_e_, expected heterozygosity; *H*_o_, observed heterozygosity; *N*, sample size; *N*_e_, effective population size; *R*_Q&G_, Queller and Goodnight (1989) relatedness; SNP, single nucleotide polymorphism.

Measures are for the historical and contemporary samples and subset “age” classes within the two contemporary samples. The analyses were carried out on two SNP data sets: a data set including the 2,218 SNPs common to both temporal samples, and a 7,730 SNP data set from the contemporary sample only.

a “Adult” refers to the subset of individuals in the contemporary sample group with a curved carapace length >65 cm; “Young” refers to the subset of individuals in the contemporary sample with a curved carapace length <35 and >15 cm.

**Figure 3:**
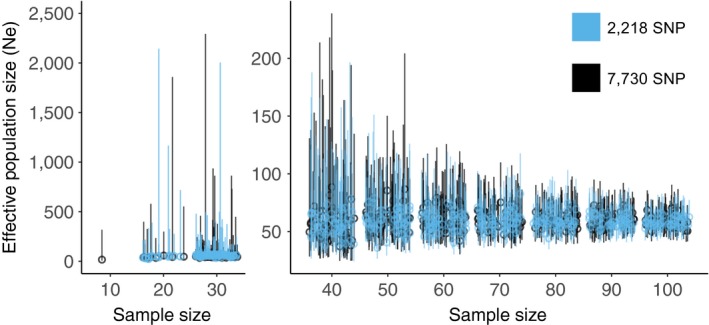
The effective population size estimates from subsets of different sample sizes taken from the pool of contemporary Pinzón giant tortoises, using a minor allele frequency of 0.05. The left and right plots are separated to allow for different scales on the *y*‐axes. For each sampling size, there were 50 subsets drawn based on the 2,218 (blue) and 7,730 (black) SNP data sets. The points are jittered, and the bars indicate 95% jackknife confidence intervals. Point estimates that were negative or infinity, or had confidence intervals that included infinity are not presented (29% of the runs, exclusively in the subsets with sample sizes of *n *=* *10–30 individuals). Thus, for example, for a sample size of 10, only one estimate is presented based on the 7,730 SNPs, and no values are presented for the 2,218 SNP data sets. SNP, single nucleotide polymorphism

The estimated *N*_e_ for the contemporary sample of Pinzón giant tortoises was 58 (95% jackknife confidence interval [CI] = 50–69; Table 1). The estimated *N*_e_ for the historical sample was undetermined (95% jackknife CI = 39.0–infinity). Simulations using subsets of different sample sizes indicated that sample sizes <30 often have very broad jackknife confidence intervals (Figure 3), often including infinity. When sampling between 40 and 100 individuals, the upper bound on the confidence interval steadily decreased, and there was little change in the estimates when using 60–100 samples for our data set. There was almost no difference in the estimate of *N*_e_ or size of the confidence interval when using the 7,730 locus or 2,218 locus data sets.

Similarly, the diversity statistics when calculated using a larger data set of 7,730 SNP loci were nearly identical for the contemporary sample group (Table 1, Supporting Information Table 3 in Appendix S1, see Supporting Information Supplementary Results in Appendix S1). Thus, the 2,218 SNP data set that overlapped with the historical sample group was used for all downstream analyses.

We found no geographical clustering of closely related contemporary adult individuals (*R* ^2^ = 0.0006, Supporting Information Figure 1 in Appendix S1). Population structure tests (i.e., STRUCTURE and DAPC) did not split the contemporary and historical samples into separate clusters (Supporting Information Figure 2 in Appendix S1). Similarly, levels of differentiation were low (but still statistically significant) between temporal samples as measured by *Phi*_ST_, and only a small proportion of loci exhibited significantly different allele frequencies (Table 2).

**Table 2:** Measures of genetic differentiation calculated between the historical and contemporary sample groups, and between “age” class subsets within the contemporary sample group

	*Phi*_ST_	DAF
Historical and contemporary	0.028^a^	0.040
Historical and adult	0.027^a^	0.037
Historical and young	0.037^a^	0.046
Adult and young	0.010^a^	0.019

Notes

“Adult” refers to the subset of individuals in the contemporary sample group with a curved carapace length >65 cm; “Young” refers to the subset of individuals in the contemporary sample with a curved carapace length >15 and <35 cm.

a Denoting significance at *p* < 0.001 for the *Phi*_*S*T_, DAF, the proportion of loci with significantly different allele frequencies (adjusted *p*‐value 0.0059).

Adult and young subsets of the contemporary sample were selected for comparison as they are at the two ends of the age continuum, representing either the early periods of the head‐start programme (“adults”) or more recent head‐start cohorts (“young”). Heterozygosity was slightly higher, and measures of inbreeding, *G*_IS_ and *F*, were more negative in the young than the adult group (Table 1). Mean relatedness among individuals within each group was equal (Table 1), with largely overlapping relatedness distributions (Figure 2b). All *Phi*_ST_ values were significant, but very low (Table 2). There was slightly greater differentiation between the young contemporary samples and the historical samples than between the adult contemporary samples and the historical samples (Table 2). These results were not influenced by the inclusion of three highly related young individuals (Supporting Information Table 4 in Appendix S1).

## 4 DISCUSSION

### 4.1 Genomic consequences of population decline and rapid demographic recovery

This study investigated changes in genomic variation in the Pinzón giant tortoise population over time, from prebottleneck to post‐head‐start programme. Using RAD‐seq and hybrid capture techniques, we genotyped 2,218 SNP loci in 25 historical and 149 contemporary individuals. We found the extent and distribution of genetic variation recovered between the prebottleneck historical and postbottleneck contemporary sample groups of the Pinzón giant tortoise were very similar. Heterozygosity was constant across temporal samples and even slightly elevated in the contemporary young sample (Table 1). No population structure was evident between temporal samples based on STRUCTURE or DAPC clustering analyses, although there were very small, but significant, *Phi*_ST_ values (Table 2). A previous microsatellite‐based study of Pinzón giant tortoises found mixed evidence in tests for a bottleneck, with no genetic signature of population decline in heterozygote excess tests, a normal distribution in the mode‐shift test indicating a stable population size, and *M*‐ratio tests suggesting a population bottleneck (Jensen, Tapia, Caccone, & Russello, 2015). Unfortunately, in this study, we were unable to explicitly test for genetic signatures of a bottleneck in the Pinzón tortoise contemporary samples given the lack of an appropriate mutation model for SNPs using the conventional heterozygote excess test (Cornuet & Luikart, 1996) and the fact that the size of our data set precluded the application of site frequency spectrum‐based methods (e.g., Excoffier, Dupanloup, Huerta‐Sanchez, Sousa, & Foll, 2013). However, our finding of similar levels of genetic diversity in the historical and contemporary samples suggests that the known demographic bottleneck has not severely impacted genetic diversity.

The mixed results in detecting a bottleneck in the Pinzón giant tortoises are in stark contrast to those from a microsatellite‐based study of a closely related species of Galapagos giant tortoise (*Chelonoidis vandenburghi*) endemic to Volcano Alcedo on Isabela Island, which revealed distinctive signatures of a population bottleneck (e.g., significant heterozygote excess, small *M*‐ratio) (Beheregaray, Ciofi, Geist et al., 2003). In that case, a major volcanic eruption approximately 74,000–120,000 years ago was inferred as the likely cause for this demographic change (Beheregaray, Ciofi, Geist et al., 2003). Based on the results of the present study, it seems likely that the bottleneck affecting Pinzón giant tortoises was not as dramatic both in terms of magnitude of size reduction and duration as the event experienced by the Volcano Alcedo tortoises.

Population genetic theory predicts a decrease in *N*_e_ due to a bottleneck (reviewed in Charlesworth, 2009; Wright, 1940). We had intended to compare *N*_e_ between the pre‐ and postbottlenecked samples, but the small sample size for the historical group (25 successfully genotyped of 78) precluded accurately estimating *N*_e_ (Table 1). Our simulations that explored the impact of sample size via downsampling of the contemporary group indicated that 50 or more individuals are required for accurate estimates of *N*_e_ (Figure 3). This conclusion follows those of England, Cornuet, Berthier, Tallmon, and Luikart (2006), who found that sample sizes equal to or greater than the true *N*_e_ are required for the linkage disequilibrium method to produce reliable estimates. Our estimated *N*_e_ in the contemporary sample was roughly the same when calculated based on the 2,218 or 7,730 SNP data sets (*N*_e_ = 58 and 59, respectively, Table 1). This value is higher than a previous estimate for Pinzón giant tortoises of *N*_e_ = 26 (95% CI = 17, 45) based on microsatellite genotypic data for 24 individuals (Garrick et al., 2015). Interestingly, conversion of the SNP‐based *N*_e_ translates to an estimated census population size (*N*_c_) of 536 based on the Frankham (1995) ratio (*N*_e_ = 0.11 *N*_c_), which more closely approximates the number of different individuals that were encountered (*n *=* *420) during a population survey conducted concurrently with our sample collection in 2014.

One persistent pattern found in all previous studies of Pinzón giant tortoises using microsatellites has been heterozygote deficit relative to Hardy–Weinberg expectations and significantly positive inbreeding coefficients (Beheregaray, Ciofi, Caccone, Gibbs, & Powell, 2003; Garrick et al., 2015; Jensen et al., 2015). Similar results were found in a recent study employing double‐digest RAD (ddRAD) sequencing to quantify genetic diversity and reconstruct population structure across all 12 extant species of Galapagos giant tortoises (Miller et al., 2018). At >26,000 SNPs, Miller et al. (2018) found significant inbreeding within the 10 Pinzón individuals included in that study. In contrast, we detected heterozygote excess in both the historical and contemporary samples, and the two measures of inbreeding we calculated are slightly negative and, in the case of *G*_IS_, not significantly different from zero (Table 1). These patterns could be due to assembly parameters allowing promiscuous mapping of reads, but we rigorously tested our assembly approach and the same pattern of slightly negative inbreeding coefficients was repeatedly found (see Supporting Information Table 1 in Appendix S1). At least one other study has found that the methodological differences between ddRAD‐seq and single‐enzyme RAD‐seq can produce different heterozygosity patterns in the same samples (Flanagan & Jones, 2018). Although the extent to which technical artefacts associated with polymorphisms in restriction enzyme cut sites may bias estimation of population genetic parameters is a matter of some debate (see Andrews et al., 2014; Puritz et al., 2014), moving forward, genome‐wide sequencing, even at low coverage (e.g., Therkildsen & Palumbi, 2017), should ameliorate such concerns.

### 4.2 Genetic legacy of the head‐start programme

The demographic recovery of the Pinzón giant tortoise species was achieved via a head‐start programme. However, this action carried the possibility of skewing genetic contributions to subsequent generations due to unequal representation of the surviving individuals. Even if all 150–200 adults on the island immediately following the bottleneck (MacFarland et al., 1974) contributed offspring to the head‐start programme, genetic diversity could have become skewed due to the over‐representation of certain families in the head‐start generations. This scenario may have occurred if some mate pairs naturally produced more offspring than others, and/or if eggs or pre‐emergent individuals were collected nonrandomly. A previous microsatellite study evaluated the genetic representativeness of the cohorts of the head‐start programme hatched in 2007 and 2009, relative to a sample of 72 adults from the wild population (Jensen et al., 2015). The conclusions were that the cohorts were genetically diverse, but that there was genetic variation in the wild adults not represented in the head‐start cohorts. Surprisingly, there were a number of alleles present in the hatchlings that were not found in the sample of adults, indicating that despite constituting a large proportion of the reproducing population, that adult sample was not fully representative of the breadth of genetic variation in the population (Jensen et al., 2015).

In the current study, we were able to more fully assess how successful the head‐start programme has been in maintaining the extent and distribution of genetic diversity by calculating pairwise relatedness among individuals in the contemporary and historical samples and comparing the distributions of relatedness values. As individuals in the historical sample were naturally reproducing, they serve as a benchmark for the typical distribution of relatedness prior to the bottleneck and establishment of the head‐start programme. We found that the distribution of pairwise relatedness in the contemporary sample matched the distribution for the historical sample (Figure 2a), indicating that overall, the head‐start programme collected eggs/individuals in a way that was not biased towards certain families. We further compared the relatedness distributions of the adult and young subsets within the contemporary sample to see whether this finding applies to both the early and recent periods of the head‐start programme and not just overall. We found largely overlapping distributions of relatedness within the two age subsets, with a slight excess of higher relatedness in the subset of young individuals (Figure 2b). Although difficult to test directly, this increase in relatedness in the young cohort may be due to an unusually large head‐start cohort collected in 2009 and reintroduced 4 years later (Jensen et al., 2015). There were some indications that the young group is more differentiated than the adults from the historical group, as there were a larger proportion of loci with significantly different allele frequencies and a slightly higher *Phi*_ST_ value (Table 2). These patterns are likely due to background levels of genetic drift operating in a small population over time. More broadly, levels of heterozygosity and inbreeding were similar between the two age class subsets (Table 1), suggesting that genetic diversity appears to have been captured consistently by the head‐start programme over a ~50 year period.

### 4.3 Insights from temporal sampling

In this study, we had the benefit of historical samples to provide context for interpreting levels of diversity observed in the contemporary population. Prebottleneck sampling of multiple individuals is unavailable for most species of conservation concern, and so indirect estimates of the severity and genetic impacts of bottlenecks must be relied upon. In some cases, the bottlenecked population can be compared to a stable population of the same species (Whitehouse & Harley, 2001) or related species (Akst, Boersma, & Fleischer, 2002; Waldick, Kraus, Brown, & White, 2002) to indirectly assess the genetic impacts of population decline. The previous archipelago‐wide studies of Galapagos giant tortoises have taken this approach and compared levels of variation in each species to gain insights into population history (e.g., Beheregaray, Ciofi, Caccone et al., 2003; Beheregaray, Ciofi, Geist et al., 2003; Ciofi, Milinkovitch, Gibbs, Caccone, & Powell, 2002; Garrick et al., 2015). However, interpreting the baseline provided by comparing Pinzón giant tortoises to other Galapagos tortoises has been complicated as *C. duncanensis* maintains higher levels of genetic variation, particularly in the mitochondrial genome, than most of the other extant species, despite having gone through a substantial bottleneck (MacFarland et al., 1974; Pritchard, 1996). In a parallel study of mitochondrial genetic variation in the temporal samples of Pinzón giant tortoises, Jensen et al. (2018) found haplotypic diversity was equal over the mitogenome, although insights from particular genes or subsets of genes were inconsistent. Here, through direct comparison of pre‐ and postbottleneck samples, it is apparent that the extent and distribution of nuclear genetic variation have been maintained through time (Table 1, Figure 2).

Although the study design used here provides unique insights, methods for collecting and analysing population genomic data from historical specimens, in the context of a temporal study, are still maturing. In Appendix 1, we detail important considerations for study design and implementation, including those related to initial SNP discovery, trade‐offs associated with balancing sample size and SNP number, and quality control.

## 5 CONCLUSIONS

Over the past several decades, considerable attention has been given to documenting the genetic consequences of population declines (Frankham, 2005; Frankham et al., 2017), yet rarely have there been opportunities to test theoretical population genetic predictions using temporal pre‐ and postbottleneck sampling or to evaluate the impacts of conservation programmes using samples from before the intervention. In the case of Pinzón giant tortoises, the harvesting of many individuals in the early 20th century for museum collections has provided an opportunity to directly assess genetic patterns associated with population decline and recovery, in this case facilitated through a head‐start programme. Given the increased capacity to mine the genome even from highly degraded sources of DNA, empirical studies using temporally spaced samples will continue to enrich our understanding of evolutionary processes and help inform conservation action.

## CONFLICT OF INTEREST

None declared.

## DATA ACCESSIBILITY

The data sets generated in this manuscript are available from the Dryad Digital Repository: https://doi.org/10.5061/dryad.7tp3sg0.

## APPENDIX 1

### Insights from temporal sampling

As methods for collecting and analysing population genomic data from historical specimens in the context of a temporal study are still maturing, we provide here some important considerations for study design and implementation. We chose to use restriction‐site‐associated DNA sequencing (RAD‐seq) for the contemporary sample to discover variable regions of the genome that could be targeted using hybridization baits in the historical samples. This procedure may have introduced some ascertainment bias, in that only RAD loci known to have variable sites in the contemporary samples were targeted. Our filtering of variable sites was designed to minimize this potential bias by selecting the first occurring single nucleotide polymorphism (SNP) in the RAD‐seq read when both contemporary and historical individuals were considered, which was not necessarily the SNP identified in the initial analysis. In our case, there were 48 SNPs retained in the final data set that were not polymorphic in the contemporary sample group, suggesting that SNPs other than those identified during the initial analysis were ultimately retained. Additionally, in the data set used for temporal analyses, we applied the minor allele frequency filter to the combined sample, to ensure that the loci retained were informative in both temporal groups.

During the filtering process for the SNP data set, a trade‐off was necessary between the number of historical individuals retained and the number of SNP loci that met filtering criteria. Here, the final data set consisting of 25 historical individuals genotyped at 2,218 SNP loci is lower than the data set initially targeted (baits were constructed for 8,918 SNPs), but is still a very encouraging outcome that demonstrates the possibilities afforded by targeted capture. Furthermore, the larger number of loci genotyped in the contemporary samples (7,730 SNPs) provided almost exactly the same diversity estimates as the subset of 2,218 loci (Table 1), lending confidence that our findings would remain unchanged with additional loci from the historical samples. The mean depths for the historical and contemporary SNP data sets were about equal, with genotyping error rates similar to what would be expected based on the coverage (Fountain, Pauli, Reid, Palsbøll, & Peery, 2016).

We were able to directly assess genotyping error rates using two pairs of contemporary samples that had been run in duplicate. Duplicate historical individuals were not included in the study design, so a similar calculation specific to that data set is not possible. Errors in genotypes can arise during data collection steps, including PCR errors during amplification, sequencing errors and postmortem DNA degradation in the case of historical samples; or during data processing, including alignment errors and filtering steps (e.g., read depths contributing to genotype calling). We expect the levels of genotyping error due to sequencing errors and data processing to be similar between the historical and contemporary data sets, as the sequencing was run using the same chemistry and instrument, and using the same data quality filters. Postmortem DNA degradation was accounted for during the data processing using MAPDAMAGE (Jonsson, Ginolhac, Schubert, Johnson, & Orlando, 2013) to rescale nucleotide quality scores suspected to be impacted by DNA degradation. Thus, in our data set, we expect the genotyping error rates calculated from the contemporary samples to be reasonably representative of error rates in the historical samples. This may be especially the case given that the historical individuals had on average greater read depth for genotypes (18.3×) than contemporary individuals (14.0×). Directly assessing genotyping error rates using duplicate samples would be preferable, however, and should be factored into the design of future studies.
