# Supplementary material for: Population genomics through time provides insights into the consequences of decline and rapid demographic recovery through head‐starting in a Galapagos giant tortoise
Source: Evol Appl. 2018 Aug 13;11(10):1811–21. doi: 10.1111/eva.12682 (PMC6231475; doi:10.1111/eva.12682)
Supplement: Supplementary file 1 [file EVA-11-1811-s001.docx]

SUPPLEMENTARY METHODS

**Sample collection and DNA extraction**

All contemporary samples were collected in partnership with the Galapagos National Park, and in accordance with the University of British Columbia animal care protocol #A14-0239. Blood samples were transported under CITES export permit #15EC000001/VS and import permit #14CA03454/CWHQ-1C. Blood samples from the contemporary tortoises were stored in tubes containing a lysis buffer (100 mM Tris–HCl, pH 8.0; 100 mM EDTA, pH 8.0; 10 mM NaCl; 0.5 % SDS; Longmire et al. 1997) and kept at ambient temperature in the field and at 4 °C upon arrival in the lab. Genomic DNA was extracted from these contemporary samples using a NucleoSpin QuickBlood kit (Machery-Nagel) following the manufacturer’s protocols, with the addition of RNaseA (Qiagen).

For the historical individual samples, a Dremel rotary tool with a cutting blade was used to initially scrape off the surface of the bone. A wedge cut was performed targeting 100–200 mg of material (mean 164 mg). Samples were stored in dry tubes at ambient temperatures until DNA was extracted. Samples were ground while submerged in liquid nitrogen using a Spex 6770 freezer mill (5 minutes precooling, 1 minute of grinding at 10x per second). Samples were demineralized in a solution of 3 mL 0.5M EDTA pH 8.0, 150 μL 10% SDS and 100 μL of 20mg/ml Proteinase K and incubated overnight at 56 °C. The lysate was concentrated to 250 μL using Amicon Ultra-4 30kDa tubes by centrifugation. The resulting 250 μL of lysate was mixed with 5x volume of buffer PB and added in three steps to a MinElute (Qiagen) column and centrifuged, removing the flow through after each step. The column was washed twice with 750 μL of PE and centrifuged, allowing desalting for 5 minutes during the first wash. The elution was performed using 50 μL of ultra-pure water preheated to 56 °C.

**Restriction site associated DNA sequencing**

RAD libraries were constructed using 500 ng of input DNA of each contemporary sample digested with the *Sbf1* restriction enzyme (New England Biolabs Inc.), and pooled into three libraries of 48 or 52 individually barcoded samples. The barcodes used were six base pairs (bp) long, and each differed by at least two bases (Hohenlohe et al. 2010, Miller et al. 2012). A sonicator (Bioruptor^®^ NGS; Diagenode) was used to shear the DNA to a mean length of ~500 bp, and automated size selection was performed using a Pippin Prep^™^ (Sage Science) to isolate DNA fragments between 350 and 600 bp. Each library was initially sequenced using one full lane of paired end 150 bp Illumina HiSeq 2500 in rapid run mode; additional sequencing of two libraries was required to achieve the target number of high quality reads.

**RAD-Seq assembly and SNP discovery**

Sequence assembly and SNP discovery were performed using the STACKS V1.3 suite of scripts (Catchen et al. 2011, Catchen et al. 2013). The *process_radtags* module was used to separate reads by barcode, remove low-quality reads and those that lacked the *Sbf1* recognition sequence, and trim reads to 140 bp. Only paired-end reads for which both mate pairs met quality checks were retained. The *clone_filter* module was used to identify and remove polymerase chain reaction (PCR) duplicates based on paired-end reads having identical sequence that, due to the random shearing during library preparation, are unlikely to have originated from separate genomic DNA molecules. After this step, only forward reads were used. The *denovo_map.pl* module was used to execute the STACKS components *ustacks*, *cstacks* and *sstacks*.

A subset of 30 individuals (10 from each library) was used to test six parameter sets. For all tests, a minimum of three identical reads were required to create a stack (-m 3), but the number of mismatches allowed between loci was varied (-M 2 or 3) as was the number of mismatches allowed between sample tags when generating the catalog (-n 1, 2 or 3). The catalogs produced by the six parameter sets had varying numbers of RAD tags, but similar numbers of variable sites, inbreeding coefficients and nucleotide diversity values (data not shown), indicating that outcomes were insensitive to choice of parameter set. We therefore used one of the middling parameter sets (-m3 -n 2, -M 2) and applied it to the full dataset. Following *denovo_map.pl*, the *rxstacks* module was run using a lnl cut off of -15, with the conf_filter and prune_haplos options turned on. The *populations* module was then run using a minimum stack depth of five, with loci required to be present in 70% of individuals, a minimum minor allele frequency of 0.05 and a lnl cut off of -30.

**Capture bait design**

The 140 bp RAD tag sequences were provided to MYcroarray (Ann Arbor, MI) for bait design, which included evaluating bait specificity. Baits were 80 bp long with 20 bp between overlapping baits (60 bp overlap, 4x bait coverage per locus). Baits were BLASTed (Altschul et al. 1990) against the *Chrysemys picta* genome (Shaffer et al. 2013) to estimate hybridization melting temperature and bait specificity. The *Chrysemys picta* genome was the closest complete genome available at the time of bait generation, but is in a different family than *C. duncanensis*; thus we used relatively relaxed stringency when assessing hybridization specificity. Baits passed filtering if they were expected to have at most 10 hits between 62.5–65 °C and four hits above 65 °C, and fewer than two passing baits on each flank.

**Historical library preparation and capture**

Each historical sample was uniquely barcoded using dual indexes as part of a blunt-end library preparation. Individual libraries were pooled in equimolar amounts prior to capture. Captures were performed on pools of four individuals using 0.5x ng of baits, which were then amplified and quantified before being pooled for sequencing.

**Capture sequence data processing and assembly**

The 340 bp long reference sequences in fasta format were indexed using BWA (version 0.7.11) *index* (Li and Durbin 2009) and SAMTOOLS (version 1.1) *faidx* (Li et al. 2009). The sequences were processed and assembled using the BAM pipeline in PALEOMIX (version 1.2.6, Schubert et al. 2014), which employs other, standard bioinformatics tools alongside native scripts to support the pipeline. Briefly, the demultiplexed fastq files from the sequencing provider were trimmed of adapter sequences and low quality/ambiguous bases using ADAPTERREMOVAL (version 2.1.7, Lindgreen 2012). As part of the trimming process, overlapping paired-end reads were merged. In the mapping stage, unmerged reads were excluded, since the insert size for endogenous historical DNA is expected to be short enough for merging (<289 bp), as recommended for historical samples in the PALEOMIX documentation. Processed reads were mapped to the reference sequences using BWA *aln* (Li and Durbin 2009) with seeding disabled. PCR duplicates were filtered using the function *MarkDuplicates.jar* in PICARD (version 2.6.0, http://broadinstitute.github.io/picard/) and *paleomix rmdup_collapsed*, a function that is part of the PALEOMIX pipeline. MAPDAMAGE2.0 (Jonsson et al. 2013) was used to rescale the quality scores of bases that were potentially the result of post-mortem DNA damage. The alignments of BAM files were further improved using GATK *IndelRealigner* (McKenna et al. 2010).

**Genotype calling and SNP filtering**

Genotype calling was performed using SAMTOOLS *mpileup* (with the settings -ug -t DP) and BCFTOOLS *call* (using -mv -f GQ; Li et al. 2009) and exported in vcf format. Filtering of the vcf files was done using VCFTOOLS (Danecek et al. 2011). Half of the historical individuals had very little data (fewer than 100,000 reads mapped), and were removed from subsequent analyses. The variable sites were filtered as follows: a minimum read depth of 6x was required, a minimum genotype quality of 18 was required, the locus had to be present in at least 50% of individuals overall, and in 50% of the retained historical individuals. All loci with a mean depth of coverage greater than two standard deviations above the mean depth were removed. RAD tags with more than 4 SNPs were discarded. SNPs were required to have a minor allele frequency of 0.05 over the whole sample. Departure from Hardy-Weinberg equilibrium (HWE) was assessed for significance assessed using the correction for false discovery rate described by Benjamini and Yekutieli (2001). Finally, we thinned the number of SNPs retaining only the first SNP in each 140 bp RAD tag. Individuals with greater than 50% missing data were removed (Supplemental data file 1).

A separate round of SNP variant detection was done using only the contemporary samples, starting from the SAMTOOLS *mpileup* step. This procedure produced a larger SNP dataset than possible from the combined data that was subsequently used for contemporary samples-only analyses and to assess whether the smaller SNP dataset provided the same signal as the larger one. Filtering was the same as above (minimum depth of 6, minimum genotype quality of 18, loci genotyped in 50% of individuals, mean depth within two standard deviations of the mean, minor allele frequency of at least 0.05, maximum of 4 SNP loci per RAD tag, meeting HWE expectations, selecting the first SNP per RAD tag).

**Other assembly and genotype calling approaches tested**

To test the impact of assembly and genotype calling software choice, PALEOMIX was also run for the contemporary samples using BOWTIE2 v2.3.2 (Langmead and Salzberg 2012) and the RADtag reference. We tested using GATK *HaplotypeCaller* to call genotypes from both the BWA and BOWTIE2 assemblies. The same SNP filtering procedure described above for the contemporary samples only was used, and inbreeding coefficients were calculated in VCFTOOLS. We also calculated genotyping error between the replicate pairs of individuals for the SNP datasets produced by each combination of software. The tests of different combinations of assembly and genotype calling software for the contemporary samples all produced datasets with roughly the same number of SNP loci retained after filtering (see Supplementary Table 1). In all cases, the mean inbreeding coefficient was slightly negative. Genotyping error ranged from 7-4%, with the lowest rate found with the combination of BWA and SAMTOOLS/BCFTOOLS, which is the method ultimately used for the datasets in this study.

**Removing highly related individuals**

To determine whether *Phi_ST_* and the proportion of alleles with significantly different allele frequencies were influenced by the inclusion of several individuals with high relatedness to others within the young group, we identified the three indivduals with the most pairwise relatedness values above 0.125. We excluded those three young individuals and recalculated *Phi_ST_* and the proportion of loci with significantly different allele frequencies.

SUPPLEMENTARY RESULTS

Initial RAD-seq libraries produced 706 million 150 bp, paired-end reads (trimmed to 140 bp) for the 150 contemporary individuals plus the two replicate samples. The mean number of retained forward reads per individual after the *clone_filter* step in STACKS was 1.7 million. The final catalogue consisted of 194,683 putative RAD loci, of which 9,890 were variable and met filtering criteria. These 9,890 RAD sequences were used for initial capture bait design, with 8,918 passing filtering and becoming part of the final set of capture targets (Supplementary data file 2). The mean merged read length for the captured sequences from the historical Pinzón giant tortoise DNA libraries was 99 bp. There was substantial variation in the number of sequenced reads for the historical individuals, resulting in 25 of 78 individuals being retained, averaging 205,000 mapped reads each after removing polymerase chain reaction (PCR) duplicates. Low frequencies of cytosine deamination were identified, occurring at <5% even at read ends.

For the combined dataset of 2,218 SNPs (Supplemental Table 2), our variant detection and filtering steps allowed for loci to be invariable in one of the two temporal sample groups; 48 and 57 loci were monomorphic in the contemporary and historical samples, respectively. The mean amount of missing data (14%) was equal in the contemporary and historical samples. Genotype calls were supported by a greater read depth on average in the historical (18.3x) than the contemporary sample (14.0x). Based on two pairs of duplicate contemporary individuals and the 2,218 locus dataset, the genotyping error rates was 4.5%.

A larger dataset of 7,730 SNP loci (Supplementary Table 3) was available for analysis in the contemporary but not the historical sample group due to missing data. Diversity summary statistics were calculated for this “contemporary only” dataset to evaluate whether genetic patterns were being adequately represented by the 2,218 loci in the combined dataset. The larger 7,730 locus contemporary only dataset had a mean genotype depth of 14.6x, 15% mean missing data, and 4.0% and 4.5% genotype error rates between the duplicate individuals, similar to the combined dataset. Diversity statistics were nearly identical for the contemporary sample, when calculated using 2,218 or 7,730 SNP loci (main text Table 1).

SUPPLEMENTARY REFERENCES

Altschul, S. F., W. Gish, W. Miller, E. W. Myers, and D. J. Lipman. 1990. Basic local alignment search tool. Journal of Molecular Biology **215**:403-410.

Benjamini, Y., and D. Yekutieli. 2001. The control of the false discovery rate in multiple testing under dependency. Annals of Statistics **29**:1165-1188.

Catchen, J., P. A. Hohenlohe, S. Bassham, A. Amores, and W. A. Cresko. 2013. Stacks: an analysis tool set for population genomics. Molecular Ecology **22**:3124-3140.

Catchen, J. M., A. Amores, P. Hohenlohe, W. Cresko, and J. H. Postlethwait. 2011. Stacks: building and genotyping loci de novo from short-read sequences. G3 (Bethesda) **1**:171-182.

Danecek, P., A. Auton, G. Abecasis, C. A. Albers, E. Banks, M. A. DePristo, R. E. Handsaker, G. Lunter, G. T. Marth, S. T. Sherry, G. McVean, R. Durbin, and G. 1000 Genomes Project Analysis 2011. The variant call format and VCFtools. Bioinformatics **27**:2156-2158.

Hohenlohe, P. A., P. C. Phillips, and W. A. Cresko. 2010. Using population genomics to detect selection in natural populations: key concepts and methodological considerations. International Journal of Plant Science **171**:1059-1071.

Jonsson, H., A. Ginolhac, M. Schubert, P. L. Johnson, and L. Orlando. 2013. mapDamage2.0: fast approximate Bayesian estimates of ancient DNA damage parameters. Bioinformatics **29**:1682-1684.

Langmead, B., and S. L. Salzberg. 2012. Fast gapped-read alignment with Bowtie 2. Nature methods **9**:357-359.

Li, H., and R. Durbin. 2009. Fast and accurate short read alignment with Burrows-Wheeler transform. Bioinformatics **25**:1754-1760.

Li, H., B. Handsaker, A. Wysoker, T. Fennell, J. Ruan, N. Homer, G. Marth, G. Abecasis, R. Durbin, and S. Genome Project Data Processing. 2009. The Sequence Alignment/Map format and SAMtools. Bioinformatics **25**:2078-2079.

Lindgreen, S. 2012. AdapterRemoval: easy cleaning of next-generation sequencing reads. BMC Research Notes **5**:337.

Longmire, J., M. Maltbie, and R. Baker. 1997. Use of 'lysis buffer' in DNA isolation and its implication for museum collections. Occasional Papers the Museum Texas Tech University:1–3.

McKenna, A., M. Hanna, E. Banks, A. Sivachenko, K. Cibulskis, A. Kernytsky, K. Garimella, D. Altshuler, S. Gabriel, M. Daly, and M. A. DePristo. 2010. The Genome Analysis Toolkit: a MapReduce framework for analyzing next-generation DNA sequencing data. Genome Research **20**:1297-1303.

Miller, M. R., J. P. Brunelli, P. A. Wheeler, S. Liu, C. E. Rexroad, Y. Palti, C. Q. Doe, and G. H. Thorgaard. 2012. A conserved haplotype controls parallel adaptation in geographically distant salmonid populations. Molecular Ecology **21**:237-249.

Queller, D. C., and K. F. Goodnight. 1989. Estimating relatedness using genetic markers. Evolution **43**:258–275.

Schubert, M., L. Ermini, C. Der Sarkissian, H. Jonsson, A. Ginolhac, R. Schaefer, M. D. Martin, R. Fernandez, M. Kircher, M. McCue, E. Willerslev, and L. Orlando. 2014. Characterization of ancient and modern genomes by SNP detection and phylogenomic and metagenomic analysis using PALEOMIX. Nature Protocols **9**:1056-1082.

Shaffer, H. B., P. Minx, D. E. Warren, A. M. Shedlock, R. C. Thomson, N. Valenzuela, J. Abramyan, C. T. Amemiya, D. Badenhorst, K. K. Biggar, G. M. Borchert, C. W. Botka, R. M. Bowden, E. L. Braun, A. M. Bronikowski, B. G. Bruneau, L. T. Buck, B. Capel, T. A. Castoe, M. Czerwinski, K. D. Delehaunty, S. V. Edwards, C. C. Fronick, M. K. Fujita, L. Fulton, T. A. Graves, R. E. Green, W. Haerty, R. Hariharan, O. Hernandez, L. W. Hillier, A. K. Holloway, D. Janes, F. J. Janzen, C. Kandoth, L. Kong, A. J. de Koning, Y. Li, R. Literman, S. E. McGaugh, L. Mork, M. O'Laughlin, R. T. Paitz, D. D. Pollock, C. P. Ponting, S. Radhakrishnan, B. J. Raney, J. M. Richman, J. St John, T. Schwartz, A. Sethuraman, P. Q. Spinks, K. B. Storey, N. Thane, T. Vinar, L. M. Zimmerman, W. C. Warren, E. R. Mardis, and R. K. Wilson. 2013. The western painted turtle genome, a model for the evolution of extreme physiological adaptations in a slowly evolving lineage. Genome Biology **14**:R28.


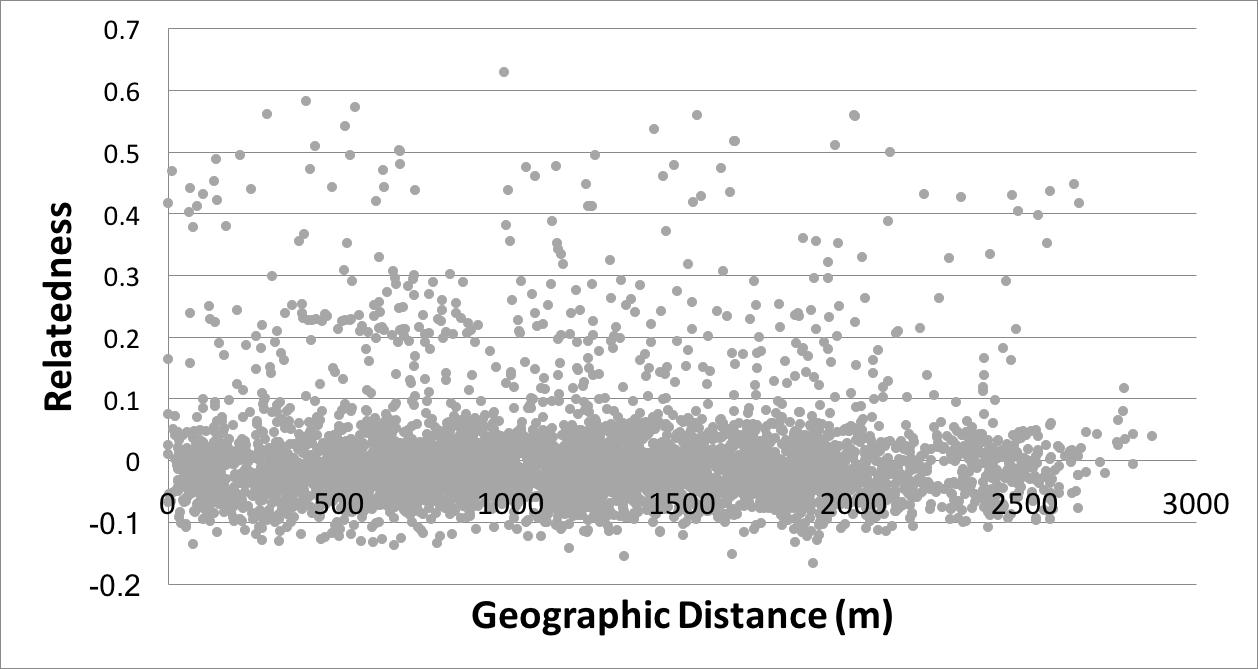


Supplementary Figure 1. Pairwise relatedness by geographic distance among individuals in the contemporary sample with a curved carapace length >50 cm. Relatedness (Queller and Goodnight 1989) was calculated from the 2,218 SNP loci.


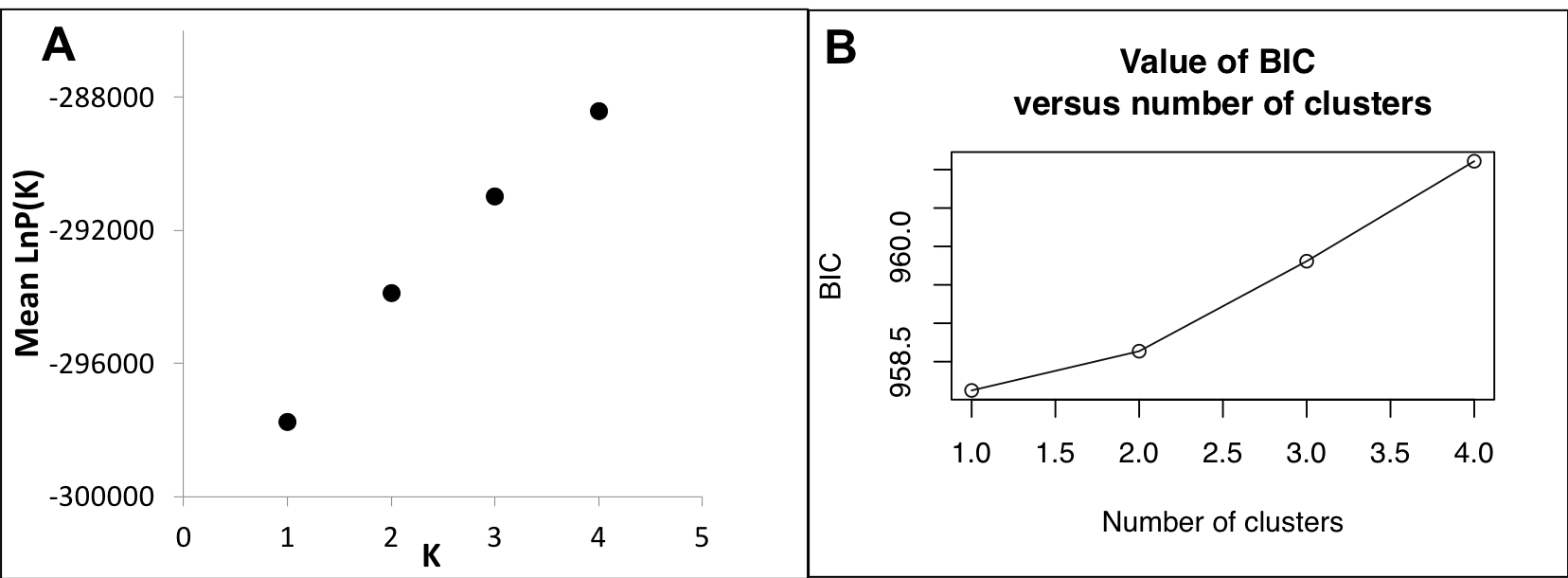


Supplementary Figure 2. Analyses to determine the optimal number of clusters (K) when evaluating the contemporary and historical sample groups together. A. A plot of ln P(*K*) for each value of *K* evaluated in the STRUCTURE analysis. B. Plot of Bayesian Information Criterion (BIC) from the DAPC analysis depicting the support for each value of *K*.

Supplementary Table 1. Comparisons of results from the different combinations of assembly and genotype calling approaches. Genotyping error rates were estimated from two pairs of duplicated individuals.

| Assembly/genotype calling methods | # SNPs after filtering | Mean F | Genotyping error |
| --- | --- | --- | --- |
| BWA/SAMTOOLS/BCFTOOLS | 7,730 | -0.08 | 4.0%, 4.5% |
| BWA/GATK | 7,588 | -0.06 | 5.6%, 6.7% |
| BOWTIE2/SAMTOOLS/BCFTOOLS | 7,678 | -0.08 | 4.0%, 4.7% |
| BOWTIE2/GATK | 7,580 | -0.06 | 5.8%, 7.1% |

*F*, inbreeding coefficient

Supplementary Table 2. Number of SNPs retained after each filtering step for the contemporary and historical combined dataset. Significance of deviation from Hardy-Weinberg equilibrium was assessed using a *P*-value adjusted for false discovery rate (Benjamini and Yekutieli 2001).

| Filtering criteria | SNP count |
| --- | --- |
| Minimum depth of 6x and genotype quality of 18 | 39,096 |
| Site genotyped in ≥50% of all individuals and ≥50% of historical individuals | 6,282 |
| Filter out over-represented loci (i.e. those with a mean depth >2x standard deviations above the mean; mean depth 22.3x, depth cut off 69.5x) | 6,007 |
| Maximum of 4 SNPs per RAD tag | 3,950 |
| Minor allele frequency 0.05 | 2,545 |
| Meeting HWE expectations (*p* > 0.0055) | 2,399 |
| Thin loci to 1 SNP per 140 bp RAD tag | 2,218 |

*SNP*, single nucleotide polymorphism; *HWE*, Hardy-Weinberg Equilibrium

Supplementary Table 3. Number of SNPs retained after each filtering step for the contemporary sample alone. Significance of deviation from Hardy-Weinberg equilibrium was assessed using a *P*-value adjusted for false discovery rate (Benjamini and Yekutieli 2001).

| Filtering criteria | SNP count |
| --- | --- |
| Minimum depth of 6x and genotype quality of 18 | 12,730 |
| Site genotyped in ≥50% of individuals | 11,980 |
| Filter out over-represented loci (i.e. those with a mean depth of 2x standard deviations above the mean; mean depth 16.3x, depth cut off 45.9x) | 11,629 |
| Minor allele frequency 0.05 | 9,512 |
| Maximum of 4 SNPs per RAD tag | 9,008 |
| Meeting HWE expectations (*p* > 0.005) | 8,586 |
| Thin loci to 1 SNP per 140 bp RAD tag | 7,730 |

*SNP*, single nucleotide polymorphism; *HWE*, Hardy-Weinberg Equilibrium

Supplementary Table 4. Measures of genetic differentiation calculated between the young group with the three individuals with the most pairwise relatedness values above 0.125 removed (Young Subset) or the full young sample (Young), and the historical and contemporary adult sample groups.

|  | *Phi_ST_* | DAF |
| --- | --- | --- |
| Historical and Young | 0.037* | 0.046 |
| Historical and Young Subset | 0.035* | 0.043 |
| Adult and Young | 0.010* | 0.019 |
| Adult and Young Subset | 0.008* | 0.014 |

* denoting significance at *p*<0.001 for the *Phi_ST_, DAF*, the proportion of loci with significantly different allele frequencies (adjusted *p*-value 0.0059).

“Adult” refers to the subset of individuals in the contemporary sample group with a curved carapace length >65 cm; “Young” refers to the subset of individuals in the contemporary sample with a curved carapace length >15 cm and <35 cm.
